# Supplementary material for: Amniotic Fluid Reduces Liver Fibrosis By Attenuating Hepatic Stellate Cell Activation
Source: bioRxiv. 2025 Jul 30:2025.02.20.639215. Originally published 2025 Feb 20. Preprint. [Version 2] doi: 10.1101/2025.02.20.639215 (PMC11870538; doi:10.1101/2025.02.20.639215)

bioRxiv preprint doi: https://doi.org/10.1101/2025.02.20.639215; this version posted July 30, 2025. The copyright holder for this preprint (which was not certified by peer review) is the author/funder, who has granted bioRxiv a license to display the preprint in perpetuity. It is made available under aCC-BY 4.0 International license.

Supplemental Figure 1. A. Liver function tests (AST and ALT) were performed on serum collected from mice at necropsy plotted as mean values (center line), with upper and lower bounds indicating the maximum and minimum values, respectively; N = 3 wild-type mice, N = 3 control mice, N = 6 CCl_4 only mice, N = 6 CCl_4+cfAF mice; $^*P < 0.05$ by Student's t-test. **B.** Results from Two-way ANOVA with multi-comparisons of mean mouse weight data for each group compared to control mice over 10-week CCl_4 animal study (accompanies **Figure 1B**). **C**. Quantification of H&E-stained murine liver histology from the CCl_4 animal study, scored blindly by an expert pathologist; N = 4 control mice, N = 4 cfAF only mice, N = 6 CCl_4 only mice, N = 5 CCl_4+cfAF mice; $^{**}P < 0.01$, $^{***}P < 0.001$, $^{****}P < 0.0001$ by One-way ANOVA with multi-comparisons (accompanies **Figure 1C**). **D.** Flow cytometry analysis of PBMC T cells, B cells, myeloid cells, or other cells (**left**) or viable splenocyte abundance (**right**) across treatment groups; N = 6 control mice, N = 6 cfAF only mice, N = 7 CCl_4 only mice, N = 5 CCl_4+cfAF mice; statistical analysis by One-way ANOVA with multi-comparisons.

Supplemental Figure 2. A. Overview of experimental design for modeling acute liver damage and cfAF administration in mice. **B.** Mean percent weight change by week, relative to starting body weight plotted by treatment group. N= 3 control

bioRxiv preprint doi: https://doi.org/10.1101/2025.02.20.639215; this version posted July 30, 2025. The copyright holder for this preprint (which was not certified by peer review) is the author/funder, who has granted bioRxiv a license to display the preprint in perpetuity. It is made available under aCC-BY 4.0 International license.

mice, N = 4 DMN only mice, N = 4 DMN+cfAF mice; $*P < 0.05$, $**P < 0.01$ by Two-way ANOVA with multi-comparisons. **C.** Liver function tests (AST and ALT) performed on serum collected from mice at necropsy plotted as mean with upper and lower bounds corresponding to maximum and minimum values, respectively; N = 3 control mice, N = 4 DMN only mice, N = 4 DMN+cfAF mice; ns indicates not statistically significant by Student's t-test. **D.** Liver sections from Control mice (top), DMN-treated mice (middle), and DMN-treated with cfAF mice (bottom) stained with Masson's trichrome; shown at 20X magnification. Scale bars (upper left) = 100 µm.

Supplemental Figure 3. A. Complete pathway analysis of RNA level changes associated with enrichment (blue) or depletion (red) of pathways in CCl_4 + cfAF mice versus CCl_4 mice (accompanies **Figure 2E**).

Supplemental Table S1. Nanostring Data

Supplemental Table S2. RNA sequencing data

Supplemental Table S3. Proteomics data

Supplemental Table S4. Phosphoproteomics data

Supplemental Table S5. Antibody information used for flow cytometry experiments.

Supplemental Table S6. Antibody information used for immunofluorescence experiments.

Supplemental Table S7. Primer sequences for PCR experiments.

bioRxiv preprint doi: https://doi.org/10.1101/2025.02.20.639215; this version posted July 30, 2025. The copyright holder for this preprint (which was not certified by peer review) is the author/funder, who has granted bioRxiv a license to display the preprint in perpetuity. It is made available under aCC-BY 4.0 International license.

Supplemental Figure 1

A.

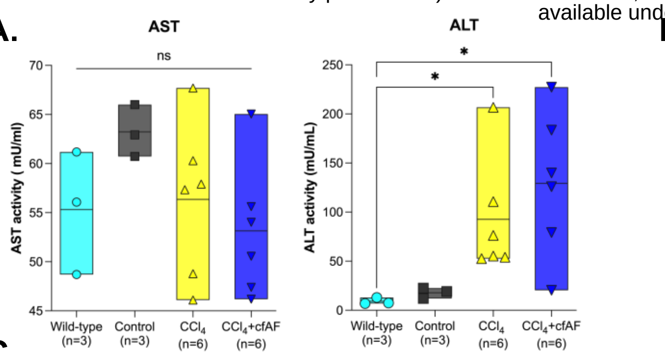

B. 10-week *P*-values

**Baseline**	***P*-value**	**Week 1**	***P*-value**
Control (n=10) vs. cfAF (n=7)	>0.9999	Control (n=10) vs. cfAF (n=7)	0.9859
Control (n=10) vs. CCl_4 (n=13)	>0.9999	Control (n=10) vs. CCl_4 (n=13)	0.2784
Control (n=10) vs. CCl_4 + cfAF (n=12)	>0.9999	Control (n=10) vs. CCl_4 + cfAF (n=12)	0.3264
**Week 2**	***P*-value**	**Week 3**	***P*-value**
Control (n=10) vs. cfAF (n=7)	0.1781	Control (n=10) vs. cfAF (n=7)	0.1893
Control (n=10) vs. CCl_4 (n=13)	0.1379	Control (n=10) vs. CCl_4 (n=13)	0.0175
Control (n=10) vs. CCl_4 + cfAF (n=12)	0.5423	Control (n=10) vs. CCl_4 + cfAF (n=12)	0.2449
**Week 4**	***P*-value**	**Week 5**	***P*-value**
Control (n=10) vs. cfAF (n=7)	0.3374	Control (n=10) vs. cfAF (n=7)	0.0468
Control (n=10) vs. CCl_4 (n=13)	<0.0001	Control (n=10) vs. CCl_4 (n=13)	<0.0001
Control (n=10) vs. CCl_4 + cfAF (n=12)	0.0046	Control (n=10) vs. CCl_4 + cfAF (n=12)	0.1239
**Week 6**	***P*-value**	**Week 7**	***P*-value**
Control (n=10) vs. cfAF (n=7)	0.0569	Control (n=10) vs. cfAF (n=7)	0.0131
Control (n=10) vs. CCl_4 (n=13)	0.0044	Control (n=10) vs. CCl_4 (n=13)	0.0002
Control (n=10) vs. CCl_4 + cfAF (n=12)	0.8482	Control (n=10) vs. CCl_4 + cfAF (n=12)	0.996
**Week 8**	***P*-value**	**Terminal**	***P*-value**
Control (n=10) vs. cfAF (n=7)	0.0002	Control (n=10) vs. cfAF (n=7)	0.0001
Control (n=10) vs. CCl_4 (n=13)	<0.0001	Control (n=10) vs. CCl_4 (n=13)	<0.0001
Control (n=10) vs. CCl_4 + cfAF (n=12)	0.2791	Control (n=10) vs. CCl_4 + cfAF (n=12)	0.024

C.

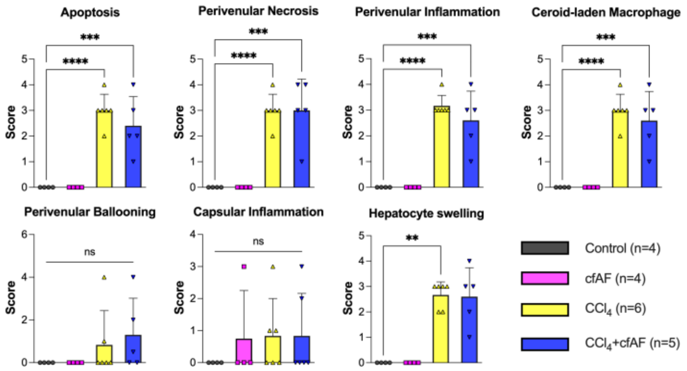

D.

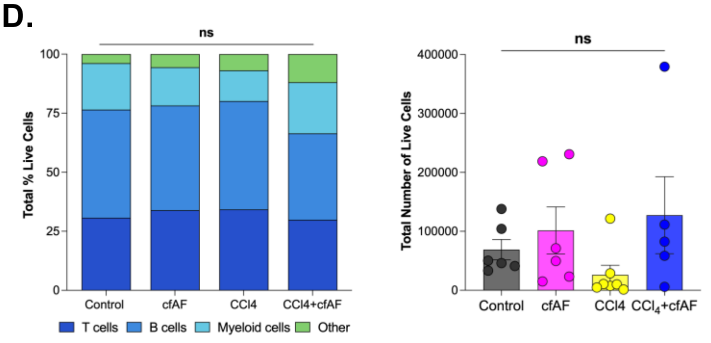

E.

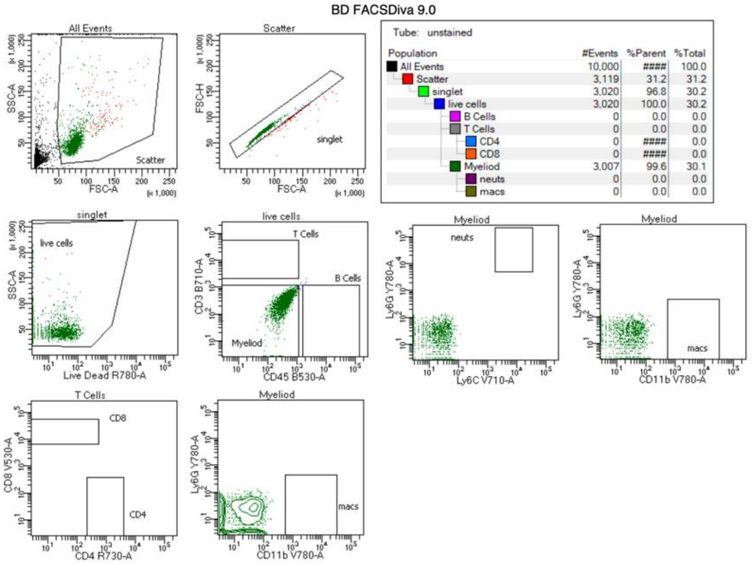

bioRxiv preprint doi: https://doi.org/10.1101/2025.02.20.639215; this version posted July 30, 2025. The copyright holder for this preprint (which was not certified by peer review) is the author/funder, who has granted bioRxiv a license to display the preprint in perpetuity. It is made available under aCC-BY 4.0 International license.

Supplemental Figure 2

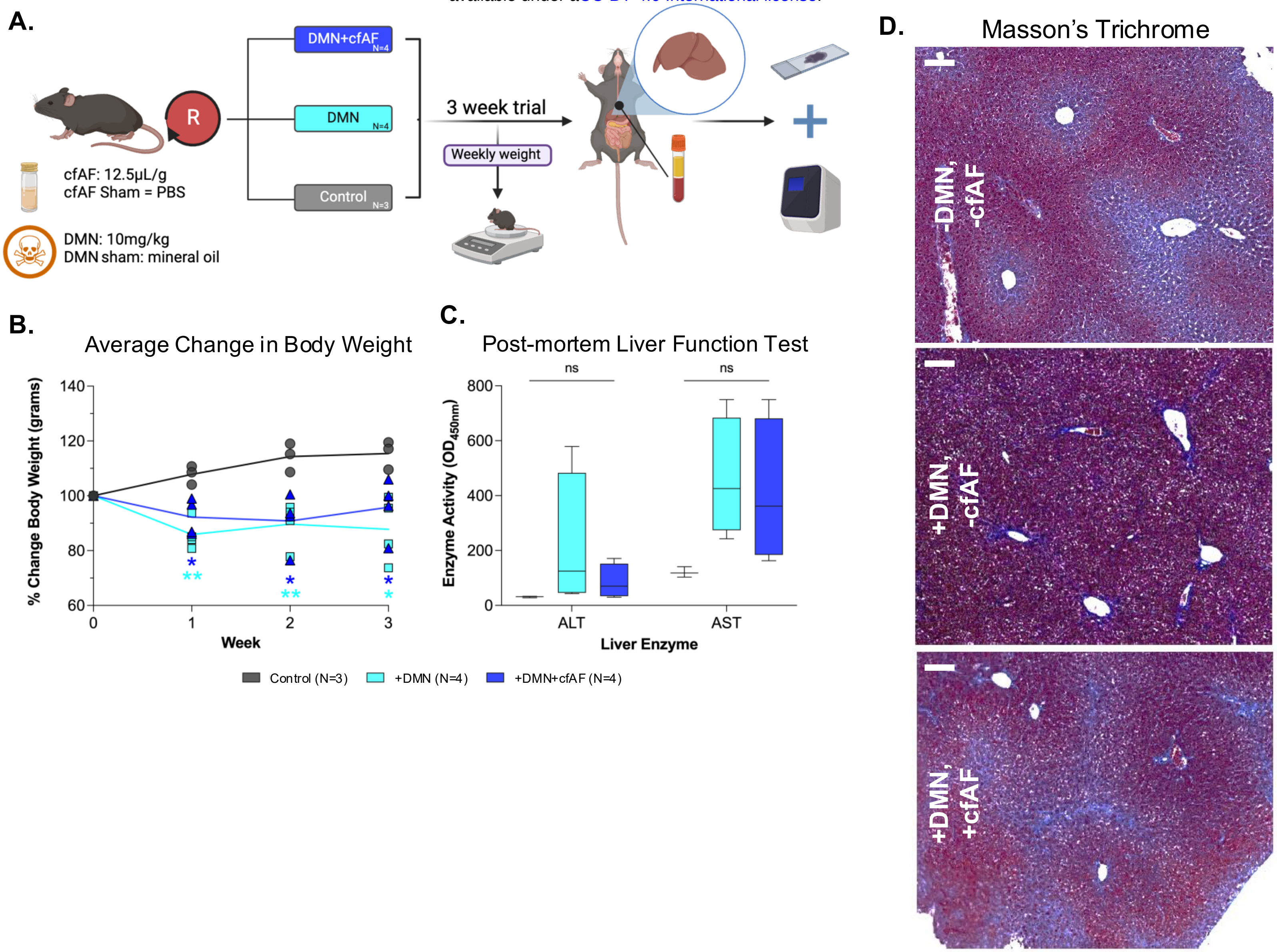

bioRxiv preprint doi: https://doi.org/10.1101/2025.02.20.639215; this version posted July 30, 2025. The copyright holder for this preprint (which was not certified by peer review) is the author/funder, who has granted bioRxiv a license to display the preprint in perpetuity. It is made available under aCC-BY 4.0 International license.

Supplemental 3

A.

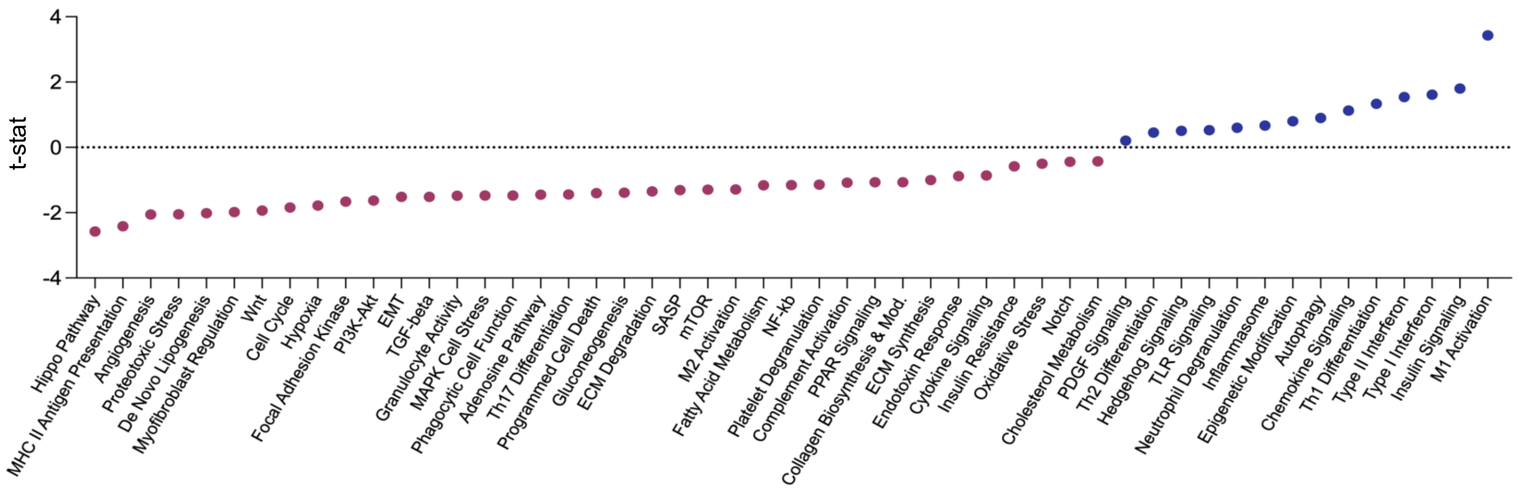

Supplement: 1 [file NIHPP2025.02.20.639215V2-supplement-1.pdf]
