# Supplementary material for: Amniotic Fluid Reduces Liver Fibrosis By Attenuating Hepatic Stellate Cell Activation
Source: bioRxiv. 2025 Feb 20:2025.02.20.639215. Preprint. [Version 1] doi: 10.1101/2025.02.20.639215 (PMC11870538; doi:10.1101/2025.02.20.639215)

bioRxiv preprint doi: https://doi.org/10.1101/2025.02.20.639215; this version posted February 20, 2025. The copyright holder for this preprint (which was not certified by peer review) is the author/funder, who has granted bioRxiv a license to display the preprint in perpetuity. It is made available under aCC-BY 4.0 International license.

Supplemental Figure 1

A.

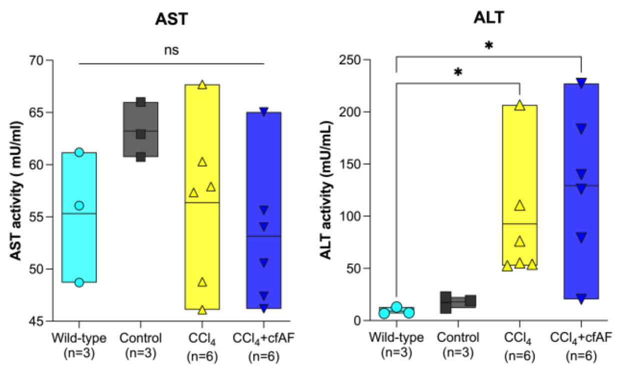

B. 10-week *P*-values

**Baseline**	***P*-value**	**Week 1**	***P*-value**
Control (n=10) vs. cfAF (n=7)	>0.9999	Control (n=10) vs. cfAF (n=7)	0.9859
Control (n=10) vs. CCl_4 (n=13)	>0.9999	Control (n=10) vs. CCl_4 (n=13)	0.2784
Control (n=10) vs. CCl_4 + cfAF (n=12)	>0.9999	Control (n=10) vs. CCl_4 + cfAF (n=12)	0.3264
**Week 2**	***P*-value**	**Week 3**	***P*-value**
Control (n=10) vs. cfAF (n=7)	0.1781	Control (n=10) vs. cfAF (n=7)	0.1893
Control (n=10) vs. CCl_4 (n=13)	0.1379	Control (n=10) vs. CCl_4 (n=13)	0.0175
Control (n=10) vs. CCl_4 + cfAF (n=12)	0.5423	Control (n=10) vs. CCl_4 + cfAF (n=12)	0.2449
**Week 4**	***P*-value**	**Week 5**	***P*-value**
Control (n=10) vs. cfAF (n=7)	0.3374	Control (n=10) vs. cfAF (n=7)	0.0468
Control (n=10) vs. CCl_4 (n=13)	<0.0001	Control (n=10) vs. CCl_4 (n=13)	<0.0001
Control (n=10) vs. CCl_4 + cfAF (n=12)	0.0046	Control (n=10) vs. CCl_4 + cfAF (n=12)	0.1239
**Week 6**	***P*-value**	**Week 7**	***P*-value**
Control (n=10) vs. cfAF (n=7)	0.0569	Control (n=10) vs. cfAF (n=7)	0.0131
Control (n=10) vs. CCl_4 (n=13)	0.0044	Control (n=10) vs. CCl_4 (n=13)	0.0002
Control (n=10) vs. CCl_4 + cfAF (n=12)	0.8482	Control (n=10) vs. CCl_4 + cfAF (n=12)	0.996
**Week 8**	***P*-value**	**Terminal**	***P*-value**
Control (n=10) vs. cfAF (n=7)	0.0002	Control (n=10) vs. cfAF (n=7)	0.0001
Control (n=10) vs. CCl_4 (n=13)	<0.0001	Control (n=10) vs. CCl_4 (n=13)	<0.0001
Control (n=10) vs. CCl_4 + cfAF (n=12)	0.2791	Control (n=10) vs. CCl_4 + cfAF (n=12)	0.024

C.

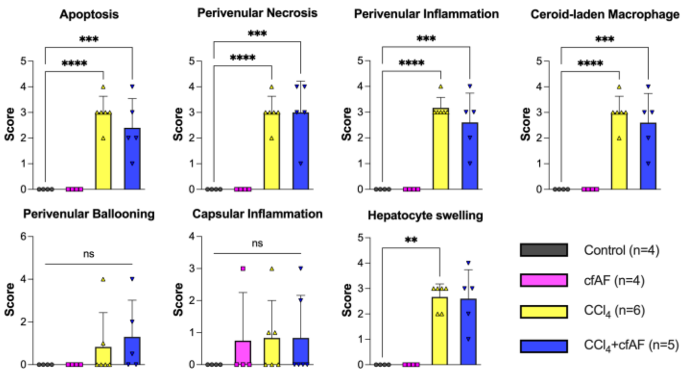

D.

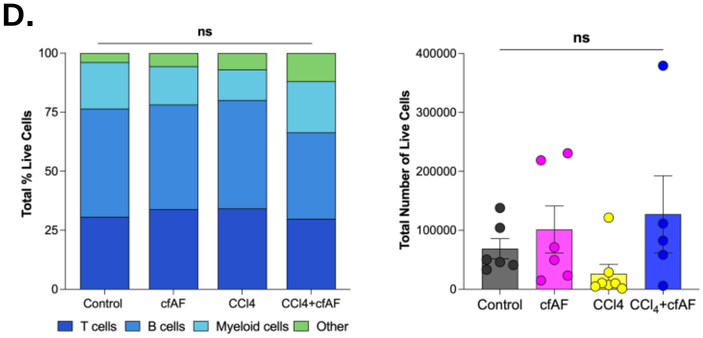

bioRxiv preprint doi: https://doi.org/10.1101/2025.02.20.639215; this version posted February 20, 2025. The copyright holder for this preprint (which was not certified by peer review) is the author/funder, who has granted bioRxiv a license to display the preprint in perpetuity. It is made available under aCC-BY 4.0 International license.

Supplemental Figure 2

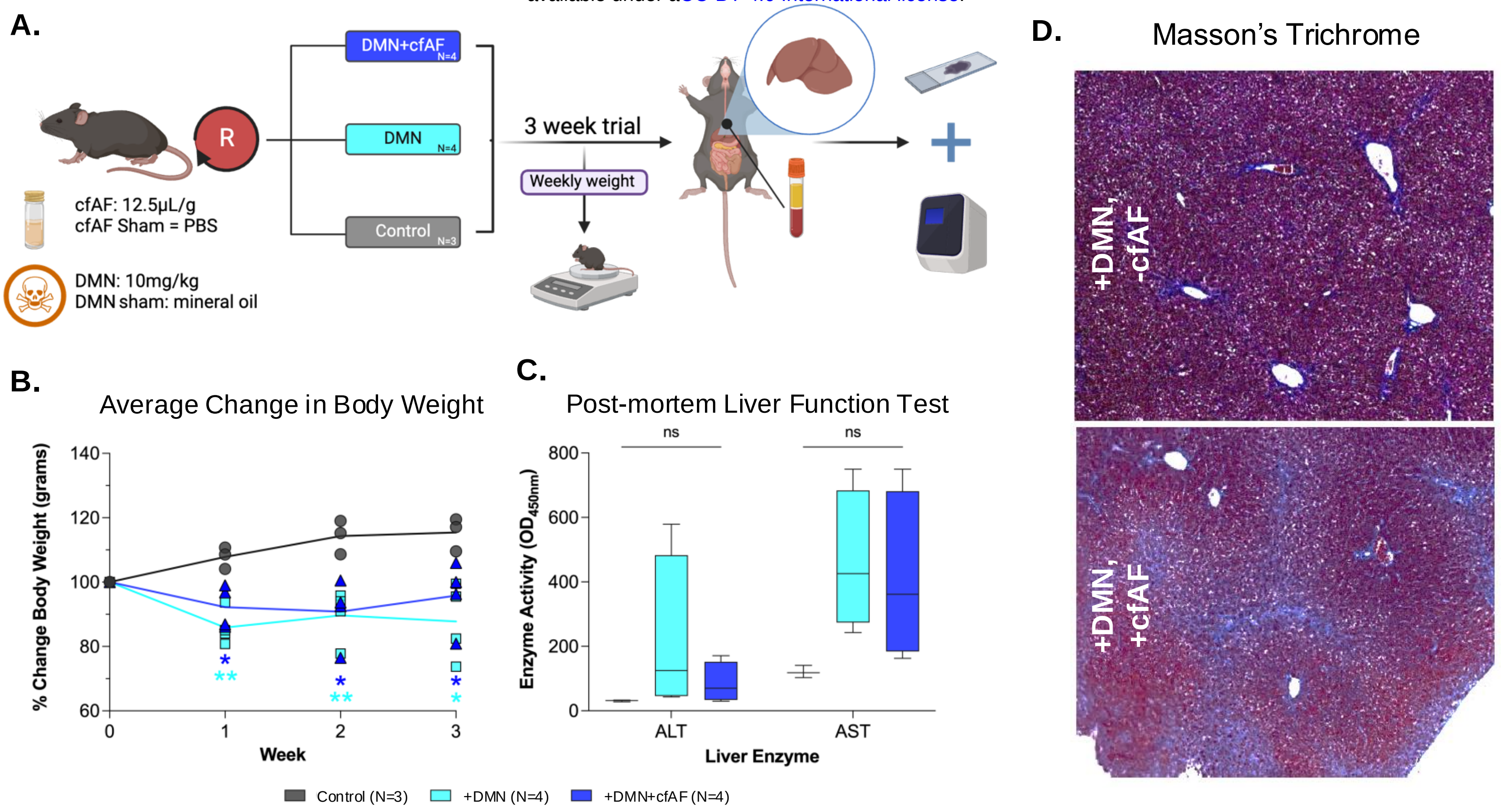

bioRxiv preprint doi: https://doi.org/10.1101/2025.02.20.639215; this version posted February 20, 2025. The copyright holder for this preprint (which was not certified by peer review) is the author/funder, who has granted bioRxiv a license to display the preprint in perpetuity. It is made available under aCC-BY 4.0 International license.

Supplemental Figure 3

A.

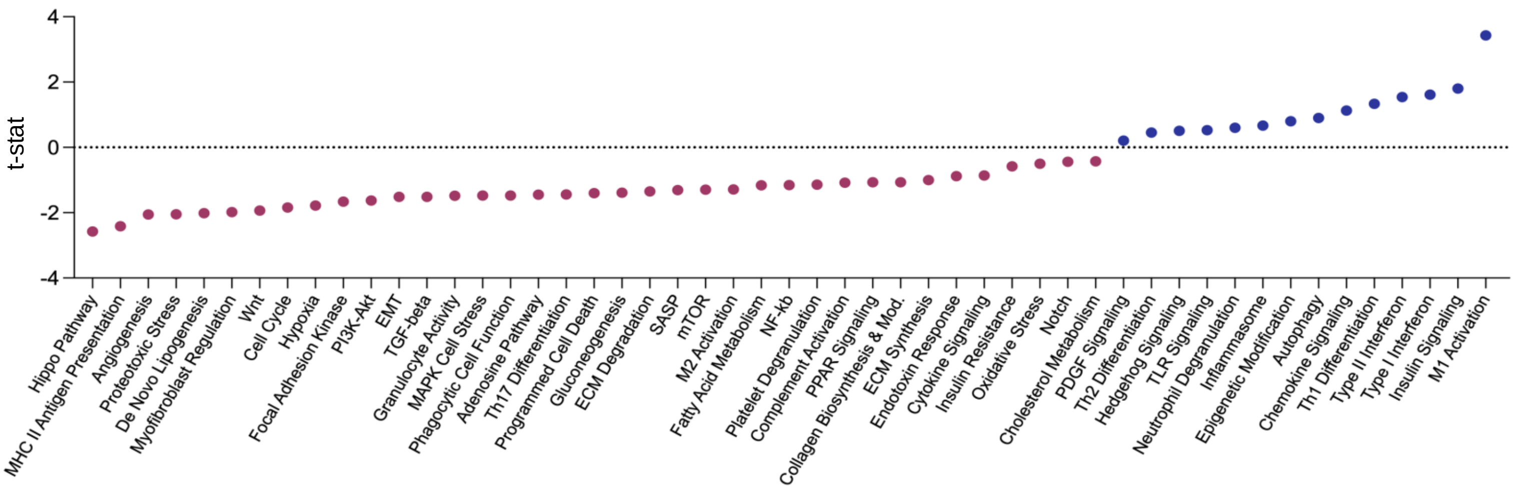

Supplement: Supplementary file 1 [file NIHPP2025.02.20.639215V1-supplement-1.pdf]
